# A proposed ethical framework for informed consent in elective robotic telesurgery

**DOI:** 10.1007/s11701-026-03304-w

**Published:** 2026-03-18

**Authors:** Catherine H. Frenkel, Marcio Covas Moschovas, Shady Saikali, Vipul Patel

**Affiliations:** 1 Division of Surgical Oncology, Department of Surgery, Levine Cancer Institute, Atrium Health, Charlotte, NC USA; 2 AdventHealth, Global Robotic Institute, Celebration, FL USA; 3 Urology Department, University of Central Florida (UCF), Orlando, FL USA; 4 Clinical Director of Experiential Education in Ethics in Surgery, IRCAD North America, Charlotte, NC USA

**Keywords:** Telesurgery, Robotics, Surgical ethics

## Abstract

Guidelines for informed consent (IC) in telesurgery are not well-defined. Ethical frameworks for clinic innovation generally focus on four key concerns, IC, safety/efficacy, patient vulnerability and conflicts of interest. The purpose of this paper is to review published recommendations on IC for telesurgery and create an ethics framework for elective telesurgical IC. A scoping literature review was performed using search terms Telesurg* AND (guideline OR consensus) in PubMed, Web of Science, and SCOPUS databases. Full text English language articles published by December 18, 2025 were included. Manuscripts were excluded if clinical telesurgery was not the subject or no formal recommendations were made regarding the IC process. A ethics framework for IC was developed based on relevant literature. 83 articles were reviewed and 4 relevant manuscripts were identified. An ethics framework for IC is proposed based on the available literature and can be further adapted regionally. A local and virtual interactive multi-stage consent process is suggested. A structured disclosure approach with key elements is outlined. Relevant terminology is defined, including implicit and explicit (situational, surgeon-specific and shared responsibility) disclosure elements. IC with disclosure of situational, local and remote surgeon-specific elements, and any shared accountability is the best practice standard for elective telesurgical consent. A multi-stage local and virtual interactive consent process should be offered in an opt-out fashion.

## Introduction

Telesurgery uses wireless networking and robotic technology to allow surgeons to operate on patients who are distantly located [1]. Although systematic reviews of telesurgical practice and the ethical concerns surrounding telesurgery have been published, the process of informed consent (IC) for telesurgery remains nebulous [2, 3]. This is because few formal guidelines for IC in elective telesurgery exist. A lack of established standards leaves patients and surgeons vulnerable to external critique. Currently, telesurgery is only practiced clinically in China [4]. Remarkably, while China has the most active clinical telesurgery program, the country has no formal guidelines available in the English literature for how IC is or should be practiced. For the rest of the global stage, telesurgery is performed on clinical trial. As such, telesurgery remains in the realm of clinical human research, with principles determined by the Declaration of Helsinki [5]. A dialogue between telesurgery physician leaders and clinical stakeholders to standardize IC is essential prior to wide-spread dissemination of this technology, so that it evolves responsibly and broad clinician and social support is maintained.

In the past 20 years, more than 30 ethics frameworks have emerged for surgical innovations [6–8]. Ethics frameworks generally apply standard ethical concerns to a new innovation and consider how the innovation may evolve in the most responsible, moral fashion. There is heterogeneity among these frameworks in relation to the way innovation is defined, the ethical issues that are raised, and the oversight mechanisms that they suggest. However, there are key ethical elements of concern that are most commonly considered by these frameworks: IC, safety/efficacy, patient vulnerability, justice and conflicts of interest (COI) [6, 9]. The purpose of this paper is to perform a scoping review of formal published recommendations from expert consensus or surgical societies that discuss IC protocols for elective telesurgery. Based on this review, the authors then propose an ethical framework for elective telesurgical IC and describe how consent can be structured to prioritize the other key ethical elements of safety, protecting vulnerable patients, principles of justice and transparency in or avoidance of COI.

## Methodology

**Fig. 1:**
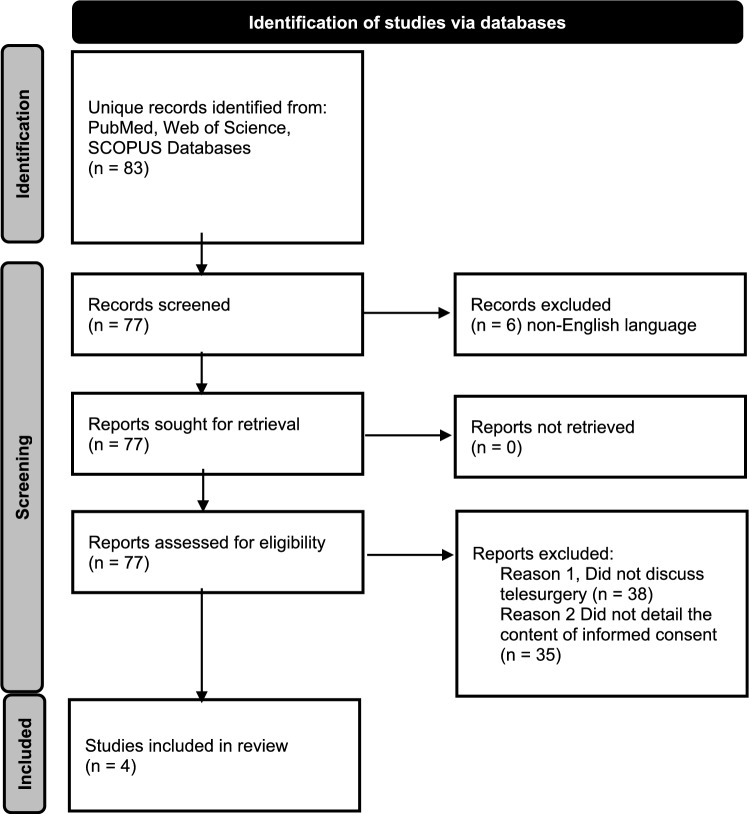
Flow diagram for article review. Source: Page MJ, et al. BMJ 2021;372:n71. https://doi.org/10.1136/bmj.n71. This work is licensed under CC BY 4.0. To view a copy of this license, visit https://creativecommons.org/licenses/by/4.0/

A scoping review of the current literature with a structured search protocol was completed by an independent reviewer. PubMed, Web of Science, and SCOPUS databases were searched for all full text articles as of December 18, 2025. Search terms included Telesurg* AND (guideline OR consensus) (Fig. 1). Manuscripts were excluded if they were not available in English or the abstract and/or manuscript subject did not meet the definition of clinical telesurgery. It this was confirmed, then the text was searched for “consent.” If the manuscript included formal recommendations regarding the process of obtaining adequate IC in human subjects, it was included in the review. References from the relevant manuscripts were also reviewed to evaluate if they met inclusion criteria. A ethical framework for elective IC is proposed based on relevant literature.

## Results

A total of 83 original articles were encountered. Six articles were not available in English [10, 11]. Notably, this included the “Guideline for remote robot surgery operation” from the Chinese Journal of General Surgery in 2025. The term “consent” did not appear in the English abstract for this manuscript. Four relevant clinical documents were identified; two from the Society of Robotic Surgery (SRS) [12, 13], one from the Japan Surgical Society [14] and another from the European Association of Urology [15] (Table 1). Similarities and differences in IC processes, disclosure, documentation and apportionment of responsibility are outlined (Table 2). All manuscripts discuss a telesurgical partnership between a local and remote surgeon for the direct benefit of the patient. Full telesurgery, during which a remote surgeon performs the procedure without a local surgeon and staff at the bedside, is not discussed in any of the articles. A fifth, earlier manuscript published in 2000 by the Society of Gastrointestinal and Endoscopic Surgeons (SAGES), entitled “Guidelines for the surgical practice of telemedicine,” is worthy of note [16]. These guidelines do not directly discuss telesurgery IC but explain that since telesurgery is “highly investigational” it “should not be performed except under IRB (Institutional Review Board) approval and by persons thoroughly familiar with the technology.” This declaration remains true to date, though technologic advancements have enhanced feasibility.

**Table 1:** Summary of articles included in scoping review

Title	Authors	Source Title	Publication Month, Year	Volume	Issue	Abstract	Relevant Sections
European Association of Urology Policy on Telesurgery	Somani, Bhaskar; Rassweiler, Jens; Liatsikos, Evangelos; Mottrie, Alexandre; Knoll, Thomas; Bedke, Jens; Bianchi, Giampaolo; Sarica, Kemal; Briganti, Alberto; Brouwers, Ton; Breda, Alberto	EUROPEAN UROLOGY	OCT, 2025	88	4	Telesurgery allows expert surgeons to operate remotely using advanced robotics. The European Association of Urology live surgery committee has created a policy to ensure that telesurgery is performed safely, with clear rules to protect patients and guide surgeons	p. 319-321: Table 1. Preoperative planning and informed consent. Section 3. Guidance for safe and ethical telesurgery. Figure 2. European Association for Urology Telesurgery Code of Conduct
Best practices in telesurgery: framework and recommendations from the society of robotic surgery (SRS) for safe and effective implementation	Patel, Vipul; Saikali, Shady; Kavoussi, Louis; Leveillee, Raymond; Albala, David; Parra-Davila, Eduardo; Rogers, Travis; Ozawa, Yu; Sharma, Rohan; Palmer, Kenneth; Marquinez, Jeff; Orvieto, Marcelo; Siddiqui, Adnan; Marescaux, Jacques; Sachdeva, Ajit; Oliva, Riccardo; Coelho, Rafael Ferreira; Rocco, Bernardo; Sighinolfi, Chiara; Roche, Martin; Martino, Martin; Ross, Sharona; Hung, Andrew; Forgione, Antonello; Abbas, Ghulam; Zhang, Xu; Aldousari, Saad; Ahallal, Younes; Alvarenga-Bezerr, Vanessa; Corder, Cathy; Moschovas, Marcio	JOURNAL OF ROBOTIC SURGERY	JUL, 2025	19	1	This framework from the Society of Robotic Surgery offers best practices for safe, effective, and ethical telesurgery. It is based on a comprehensive literature review, Delphi consensus process, and an international meeting in Orlando, Florida, in February 2024, involving experts from a variety of domains including surgery, engineering, telecommunications, hospital administration, law, and regulatory affairs. Key areas covered include the global landscape and new models of remote surgical care, technological needs like connectivity and cybersecurity, safety standards, training and credentialing for surgical teams, and scalable implementation models. It also addresses legal and ethical issues, clinical indications, and regional and international regulatory comparisons. This framework aims to be a key reference for institutions, surgical societies, policymakers, and technology developers to promote safe, equitable, and sustainable telesurgery worldwide	p. 8-9: Legal and ethical considerations. Sections on patient autonomy, informed consent, and liability disputes
International multispecialty consensus statement and expert opinion of best practices in telesurgery	Patel, Vipul; Collins, Justin W.; Marescaux, Jacques; Dohler, Mischa; Saikali, Shady; Dasgupta, Prokar; Reddy, Sumeet; Gamal, Ahmed; Patel, Ela; Rogers, Travis; Siddiqui, Adnan; Breda, Alberto; Mottrie, Alex; Hassan, Ameer; Hung, Andrew; Secord, Angeles; Rocco, Bernardo; Pugh, Carla; Sundaram, Chandru; Sighinolfi, Maria Chiara; Ellison, E. Christopher; Davila, Eduardo Parra; Wilson, Erik; Balkhy, Husam; Kaouk, Jihad; Liang, Cui; Kavoussi, Louis R.; Roche, Martin; Martino, Martin.; Anvari, Mehran; Sylla, Patrice; Coelho, Rafael Ferreira; Thomas, Raju; Clayman, Ralph; Leveillee, Raymond; Estape, Ricardo; Goldberg, Ross; Madder, Ryan; Horgan, Santiago; Magnuson, J. Scott; Nathan, Senthil; Ross, Sharona; Costello, Anthony; Xu, Zhang; Chauhan, Sanket; Redan, Jay; Satava, Richard; Miles, Brian; Sachdeva, Ajit K.; Moschovas, Marcio Covas	JOURNAL OF ROBOTIC SURGERY	APR, 2025	19	1	No abstract available	p. 6: Table 3. Ethical considerations involved in practicing. p. 9: Table 6. Delphi consensus and the synthesis of the 10 commandments of the ethical and safe exploration of telesurgery
Clinical practice guidelines for telesurgery 2022	Mori, Masaki; Hirano, Satoshi; Hakamada, Kenichi; Oki, Eiji; Urushidani, Shigeo; Uyama, Ichiro; Eto, Masatoshi; Ebihara, Yuma; Kawashima, Kenji; Kanno, Takahiro; Kitsuregawa, Masaru; Kinugasa, Yusuke; Kobayashi, Junjiro; Nakamura, Hiroshige; Noshiro, Hirokazu; Mandai, Masaki; Morohashi, Hajime Morohashi, Hajime	SURGERY TODAY	AUG, 2024	54	8	Telesurgery is expected to improve medical access in areas with limited resources, facilitate the rapid dissemination of new surgical procedures, and advance surgical education. While previously hindered by communication delays and costs, recent advancements in information technology and the emergence of new surgical robots have created an environment conducive to societal implementation. In Japan, the legal framework established in 2019 allows for remote surgical support under the supervision of an actual surgeon. The Japan Surgical Society led a collaborative effort, involving various stakeholders, to conduct social verification experiments using telesurgery, resulting in the development of a Japanese version of the “Telesurgery Guidelines” in June 2022. These guidelines outline requirements for medical teams, communication environments, robotic systems, and security measures for communication lines, as well as responsibility allocation, cost burden, and the handling of adverse events during telesurgery. In addition, they address telementoring and full telesurgery. The guidelines are expected to be revised as needed, based on the utilization of telesurgery, advancements in surgical robots, and improvements in information technology	p. 823-826 : Sections 2.2.3.3. Preoperative explanation to patients and obtaining, 2.3. Apportionment of responsibility for telesurgical support, 2.4. Patient-physician relationship in telesurgical support. 3.2.2.2. Explanation to the patient and consent acquisition. 3.3 Apportionment of responsibility for telementoring. 3.4. Patient-doctor relationship in telementoring

**Table 2:** Summary of consent elements in reviewed articles

Title	Consent Provider(s)	Timing/ Staged Consent	Disclosure Elements	Remote Surgeon-Patient Preoperative Contact	Liability/Accountability Explanation Requirements	Documentation Requirements
European Association of Urology Policy on Telesurgery (2025)	Local and remote surgeons	Patients must be asked well in advance for their permission to undergo telesurgery	General explanation of the telesurgery procedure plus 1. Risks 2. Benefits 3. Alternatives 4. Mechanism of handling complications by the local team, e.g. conversion or robotic continuation 5. Conflicts of interest of all participating team members	Virtual or online contact should be arranged between the remote surgeon and the patient before surgery for the purposes of informed consent	Designation of intraoperative roles of the local and remote surgical teams. The role and responsibility of the local team in managing complications. A predetermined protocol for immediate postoperative care must be followed under the direction of the local surgeon and team. The telesurgeon must be informed of and, where possible, involved in all decisions about postoperative patient care. The telesurgeon must be kept informed of all deviations from the care plan	1. The standard consent for surgery used by the hospital 2. Explicit consent about the remote nature of the surgery. 3. Patients should give specific consent to a live link during the operation
Best practices in telesurgery: framework and recommendations from the society of robotic surgery (SRS) for safe and effective implementation (July 2025)	Local surgeon.	The patient must be free from coercion and allowed sufficient time to consider the advantages and risks of telesurgery before deciding. In non-urgent cases, one final verbal consent can be obtained from the patient by the remote surgeon prior to the start of the procedure along with the standard documented consent	Adequate and detailed information about the procedure plus 1. Risks 2. Benefits 3. Alternatives 4. Role of remote and local surgeons 5. Plan for technical or network failure 6. Privacy concerns 7. Licensing and jurisdiction 8. Limitations of local surgeon 9. Chain of responsibility 10. Limited remote surgeon postoperative responsibility 11. Probability of successful remote completion	Efforts should be made to implement remote surgeon patient contact such as: virtual preoperative consultations or recording personalized video messages by the remote surgeon for patients to view	Clear confirmation of the designated local surgeon who will assume responsibility if telesurgery is unsuccessful or discontinued. Discussion should take place regarding post operative care and contingency plans if complications cannot be managed by the local surgeon	1. The informed consent process must be distinct from the regular surgical consent and must address specific concerns that can arise in telesurgery procedures. This should include a comprehensive list of technical, procedural, and data security risks that can be encountered
International multispecialty consensus statement and expert opinion of best practices in telesurgery (April 2025)	Local surgeon	N/A	Clear, truthful, and accessible information in a manner that is understandable and free from coercion about 1. Risks 2. Benefits 3. Capabilities of local team 4. Limitations of the local surgeon 5. Economics 6. Chain of responsibility 7. Who will complete the procedure locally 8. Likelihood of remote surgery completion	N/A	Define and communicate the shared responsibility and liability among all parties involved in robotic telesurgery, including the local and remote surgical teams and hospitals, device manufacturers, and telecommunication provider. Contingency plans should be present if complications arise that local surgeons are not capable of managing	1. Explicit consent about the remote nature of the surgery and the teams involved
Clinical practice guidelines for telesurgery 2022 (2024)	Local surgeon	Consent must be obtained in advance to share personal information with a remote team	General overview of telesurgical support plus 1. Advantages 2. Possible disadvantages 3. Information about the remote surgeon	N/A	The local surgeon and the local facility administrator are responsible for the overall outcomes of the practices related to telesurgical support and postoperative complications. The remote facility administrator should determine whether responsibility for each case will be divided (if “yes,” the nature and extent of the division must be determined). The local surgeon must be treating the patient directly as the attending physician or equivalent. The remote surgeon may participate in telesurgical support without having been involved in prior direct patient care	1. The local surgeon, local surgical staff, and remote surgeon must hold a conference in advance to thoroughly discuss the appropriateness of providing telesurgical support (patient condition, indication, surgical procedure, etc.), as well as the division of roles between the local and remote surgeons, the possibility of changing the surgical procedure, and what to do if it becomes difficult to perform the procedure as a telesurgery. The details of these discussions must be documented in the patient’s medical record. 2. Local/remote surgeons and local surgical staff should prepare and share an “Emergency Response Manual,” in advance, to deal with intraoperative emergencies. The manual must include the criteria for decision to suspend or discontinue telesurgical support, as well as the criteria for conversion to open or thoraco/laparoscopic surgery. 3. The administrator must prepare a written agreement or a written record of apportionment of perioperative responsibility

**Fig. 2:**
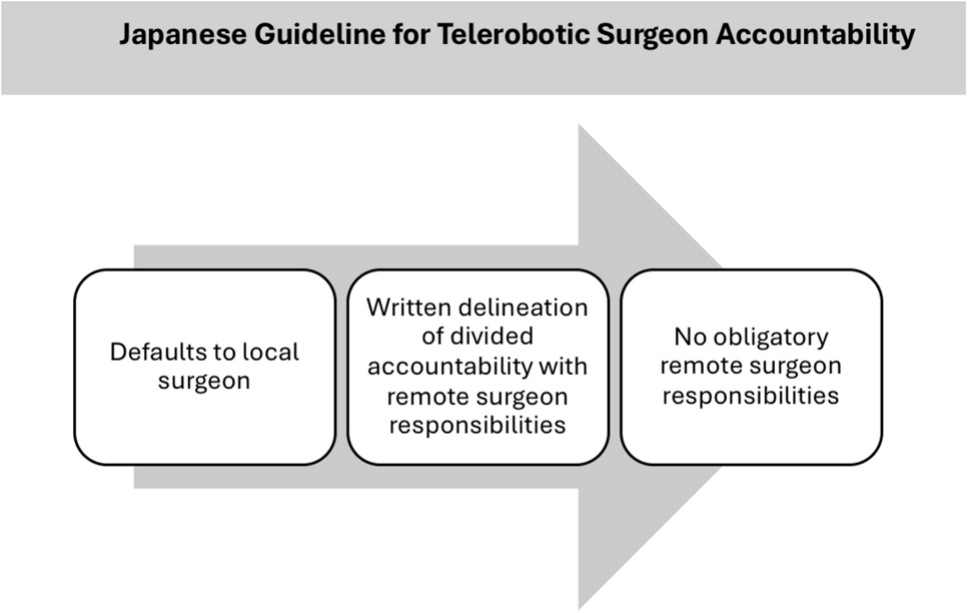
Japan surgical society apportionment of surgeon accountability

First, the Japan Surgical Society published a “Clinical practice guideline for telesurgery” in 2022. This guideline specifies that the local surgeon or a physician at the local surgical site must consent the patient. A procedural and peri-procedural overview, advantages and disadvantages must be disclosed. Information about the remote surgeon should be disclosed, but exact elements are not specified. Legal liability for complication management is not required to be discussed during IC. However, the guidelines set a default for apportionment of individual surgeon clinical responsibilities. The local surgeons and facilities are responsible for the overall outcomes. If the responsibility is to be divided between the local and remote surgeon, the nature and extent of the remote surgeon’s responsibilities must be delineated in a written record prior to surgery (Fig. 2).

Next, the SRS released two clinically relevant manuscripts in 2025. An initial manuscript entitled, “International multidisciplinary consensus statement and expert opinion of best practices in telesurgery,” which was followed by the “Best practices in telesurgery” framework, published shortly thereafter. A section of the early consensus statement is dedicated to ethical considerations for IC. Important ethical elements to include in elective telesurgical IC are outlined. These elements include reviewing the surgical chain of command if telesurgery fails or is aborted, an explanation of what procedural elements will be performed remotely, and clear discussion/documentation of surgeon clinical perioperative responsibilities. Telesurgery IC is intended to be separate from the standard surgical procedural consent, each serving a distinct purpose. Discussion of responsibilities and contingency plans to manage complications is encouraged, particularly if a complication can arise that the local surgeon would find challenging to address. Ethical concerns related to preservation of the surgeon-patient relationship, ensuring adequate disclosure and appropriate liability are also listed. A collaborative effort to bring together experts with varying perspectives to navigate telesurgery’s complex ethical, technical and legal landscape is emphasized.

The SRS updated its framework for “Best practices in telesurgery” in July 2025. Newly introduced points include the idea that the remote surgeon obtains “one final verbal consent” prior to procedure start, in addition to the standard documentation. The detailed information provided to the patient should include the probability of successful case completion using remote techniques. Sufficient time for the patient to consider the risks and advantages of surgery before consent should be allotted, although an adequate timeframe is not explicitly proposed. Video as well as written resources that review the risks and benefits of telesurgery are encouraged.

Finally, the European Association of Urology published a policy statement to address live telesurgery during urology meetings at the end of 2025. This policy adds two unique elements to consider during elective telesurgery IC [15]. First, if any non-essential party is to view the telesurgery as a “live” example for educational purposes, specific disclosure and consent must be obtained. Second, it uniquely states that virtual or online contact should be arranged between the remote surgeon and the patient before surgery for the purposes of IC. However, details to further operationalize this recommendation are not provided.

## Discussion

Telesurgery is unique in that it is a resource intense high-stakes surgical intervention with a moral purpose aimed at minimizing limitations in patient access to high-quality surgical care. Its classification stage in the IDEAL framework is between Stage I and IIa, with well-documented proof of concept, utilization limited to a few key innovators, and evolving procedure regulation and development [17]. Based on the stage of this innovation, ethical oversight in addition to research oversight is imperative [9]. The formation of a multidisciplinary safety overview group and/or a clear ethics plan will be integral for responsible evolution of this technology. During the transition from IDEAL stage I to IIa, careful attention to the IC process, not only through written documentation but also mindful stewardship of the integrity of the interaction itself will be essential [9].

The primary focus of this article is to assess and suggest the most ethical strategies for surgeons to engage in an IC discussion with patients regarding telesurgery. Regulating safe telesurgery is outside the scope of this article but technical guidelines have been published [13, 18]. Key issues such as connectivity reliability, latency, contingency planning, and cybersecurity must be sufficiently guaranteed, monitored and enforced. These operational realities directly affect patient safety and play a central role in determining ethical telesurgery practice. It should also be emphasized that the social, regulatory and legal infrastructure required to support ethical telesurgery in the United States and over geopolitical borders has yet to be built [3]. Credentialing of telesurgeons has not been standardized. With respect to international telesurgery, medical or legal governing bodies have no enforcement jurisdiction over physicians licensed abroad. International data privacy agreements do not exist. These legal and regulatory barriers have ethical implications.

The following proposed ethical framework for IC will confront implementation challenges due to various jurisdictional legal documentation requirements, language translational nuances, cultural variation that influences medical decision-making and patient vulnerability, and different local government enforcement capabilities. Without a way to enforce international ethical and legal standards, these standards have unclear power. Nevertheless, despite much that remains unknown and uncharted ground as telesurgery evolves, it remains the responsibility of the surgical community to ensure that the IC process is subject to intense ethical scrutiny. Patients and surgeons alike want confidence that telesurgery IC has universal ethical safeguards in place as it transitions from institutional regulatory oversight bodies to clinical practice. These ethical safeguards can be structurally built into the IC process. IC documentation alone is insufficient to ensure that a meaningful clinical interaction takes place when the consent is signed.

Institutions have not provided detailed summaries of their telesurgery research consent process in the published literature. A recent systematic review of all published clinical studies reporting on telesurgery is available for reference [2]. High volume centers can provide meaningful site-specific practical insights into elective IC practices, such as that described by the laparoscopic assisted robotic telepresence surgery (ARTS) program at McMaster University. Anvari et al. describes “the Canadian experience” for telementoring at McMaster University [19]. Patients signed two separate consent forms, one for the operation and one for the telerobotic intervention by a remote surgeon. Written disclosures included documented strategies planned to deal with telesurgery technical malfunctions. Transparency regarding the disclosure frameworks used in clinical telesurgical research consent will be a valuable tool to ensure ethical deployment of this technology since it transcends geopolitical borders and particularly strains traditional language, cultural and legal barriers [20].

**Fig. 3:**
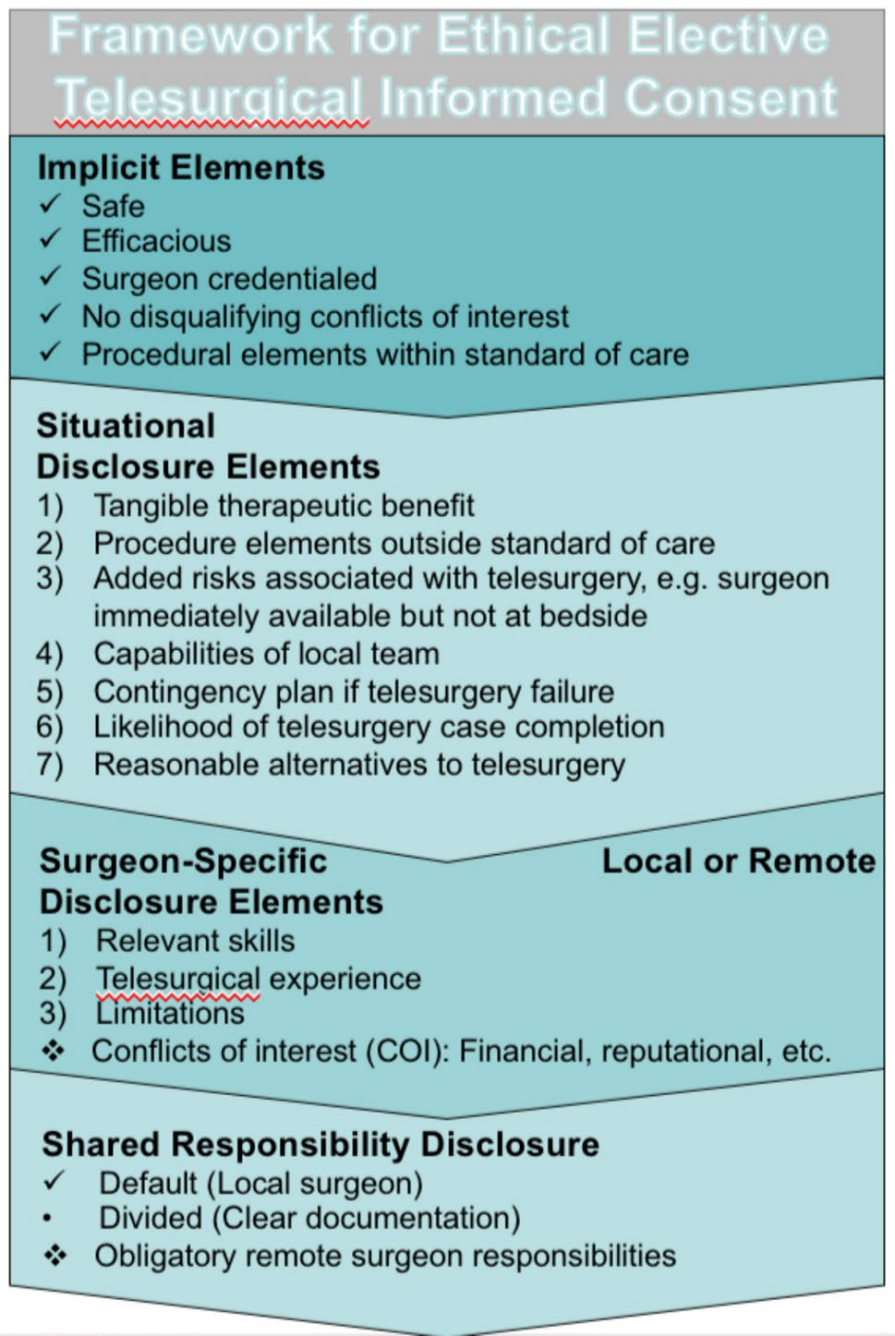
Proposed model for multi-center informed consent

Ultimately, a transparent, honest and practical ethical framework for elective telesurgery IC protects surgeons, institutions and patients. It will ensure telesurgical teams stay true to the core foundational principles of biomedical ethics – respect for autonomy, non-maleficence, beneficence and justice) [21]. The author proposes the first such ethics framework of its kind for elective telesurgery (Fig. 3). Unique to this framework for ethical IC for this surgical innovation is the division of IC disclosure elements into implicit and explicit elements. Implicit disclosure elements are well-accepted standards of disclosure for ethical IC in surgical innovation. Early in the IDEAL stages, these elements will be most consistently disclosed despite the potential impact of geopolitical/jurisdictional/regulatory divergent policies within the global surgical landscape. Once any telesurgery IC contract is entered, it should be ethically presumed that these implicit elements (safety, efficacy, adequate credentials, standard of care procedure, etc.) will be upheld unless it was clearly stated otherwise.

Contrary to standard IC, for which safety is often implied, surgeons should clearly discuss safety during IC for telesurgery both on clinical trials and as the process matures through IDEAL stages and enters mainstream practice. This is because telesurgery introduces a markedly increased level of risk, far exceeding that of non-remote surgery. Telesurgery outside of clinical trials should be safer for patients than existing reasonable alternatives to be justifiable. The onus is on the surgeon to thoughtfully communicate to the patient what significant threshold of added safety telesurgery offers the patient to offset this increased risk.

This critical ethical safeguard for clear convincing evidence supporting patient safety has not been necessary in standard robotic surgery IC. Standard robotic surgery has evolved in the United States, as well as in other countries, under more utilitarian principles. A surgical robot is an instrument surgeons use at their discretion because the benefits of this minimally invasive technology are generally well-accepted and the added risk to the patient is relatively low. Surgeon preference and longevity are factored into robotic surgery risk-benefit analyses. Social justice is not often part of this equation. Failure to demonstrate a clear threshold in operation safety along a complex robotic learning curve has, in some cases, engendered criticism of surgeons for rapid, early technology uptake with equivocal or worse safety outcomes compared to laparoscopy [22]. Negative critiques of existing clinical robotic outcomes can actually strengthen arguments favoring telesurgery, teleproctoring and telementoring, as these processes can facilitate patient safety along the steep learning curve in robotic surgery. Telesurgical IC should consistently include either a formal disclosure of either clinical equipoise regarding safety (a genuine uncertainty of risk) or a clear, quantifiable tangible therapeutic benefit. [23] Self-regulation in monitoring and reporting outcomes to support either of these disclosure statements over time will be essential.

Telesurgery is a surgical delivery system and, as it is the primary investigational element for the time being, the surgical procedure itself should remain within the standard of care, which has well-documented safety and efficacy. Credentialing is essential, based on a poll of experts [12, 13]. Surgeons must assure patients of safety and efficacy using clear, easy to understand language, e.g. the institution, surgeon, participating industry and regulatory bodies acknowledge that the minimum criteria for safe telesurgery are met. They certify that, to the best of their knowledge, the procedure being offered is reasonably safe and highly likely to be completed remotely. Safe, effective telesurgery depends on a series of complex and critical technical elements – connectivity, latency, and cybersecurity, as well as procedural check points. A checklist for safe telesurgery set-up, planning and execution has been developed by Collins et al. [24]. These technical elements and procedural checkpoints are too complex for most patients to fully comprehend during the IC process, though they do have significant potential to introduce patient risk in the event of malfunction or inadequate contingency planning. Details of these technical and procedural aspects are critical in the regulatory, legal and clinical trial realms for ethical telesurgery IC. During verbal telesurgery IC, a broad overview of how the remote and local surgeons guarantee safety and efficacy should be painted for the patient using a plain language summary of key elements and possibly multimedia educational adjuncts or decision aids that meet international standards [25].

Contrary to the implicit elements of disclosure, explicit elements of disclosure will reflect global variability, particularly in the early IDEAL innovation stages. Disclosure of situational elements is universally critical, but essentially unique and tailored to the individual clinical context and the institutions involved. Surgeon-specific and shared responsibility are universally important disclosure principles, but the nature of their disclosure will be significantly impacted by local regulatory and legal conditions. This heterogeneity is reflected by the existing published recommendations, which have regional differences with respect to surgeon-specific and shared responsibility disclosure. For example, the Japan Surgical Society guidelines suggest that only the local surgeon discloses situational or surgeon-specific elements. The remote surgeon has no disclosure responsibilities and therefore no physician-patient relationship to develop [14]. A problem with this structure of disclosure is that it erodes the integrity of the patient-surgeon relationship and exposes the patient to significant vulnerability. The major advantage of telesurgery is its potential to address rural and global disparities in surgical access, and as such, an inherent skill differential exists between the local surgeon and remote telesurgeon. This skill differential may be wide, as the learning curve is particularly steep for robotic surgery. For example, while competency in robotic colorectal surgery may be achieved after approximately 13 cases, true mastery of the skill requires upwards of 85 to over 100 cases [26]. The SRS consensus explicitly acknowledges this skill differential, documenting that 90% of experts agree it is necessary for local surgeons to fully disclose the limits of his or her expertise to the patient [12]. As societies globally collaborate to work towards a model of telesurgery consent with local adaptability, it will be important to incorporate IC counseling opportunities with remote surgeons. Disclosure will have qualitative differences when discussed by surgeons with varying experience levels.

An important unanswered ethical concern for the future is the extent to which remote telesurgeons should have obligatory postoperative responsibilities. Some guidelines, such as that of the Japan Surgical Society, set a default for apportionment of individual surgeon clinical responsibilities (Fig 2). The local surgeons and facilities are responsible for the overall outcomes. Clear preoperative written agreements must disclose if the responsibility is to be divided and the nature/extent of the remote surgeon’s responsibilities. As societies globally collaborate on telesurgery IC, any obligatory remote surgeon responsibilities must be considered.

Within the technologically advanced landscape of telesurgical innovation, the potential for virtual patient consultation with their remote surgeon is ethically meaningful and practically feasible (with translator services as necessary). A virtual interactive opportunity between patient and remote surgeon limits patient vulnerability to the local surgeon’s potential financial and non-financial (reputational benefit, recognition, excitement, etc.) COI. It bolsters, rather than degrades, the integral physician-patient relationship. Indirect physician-patient relationships are currently controversial, but there is professional and emotional value in their creation. These indirect relationships reinforce, rather than compromise, the bond between the local surgeon and patient. Local surgeons are empowered by better collegial support as they adopt new approaches or take on complicated cases [27]. Ultimately, this strategy strengthens local medical communities and optimizes their ability to deliver high-quality surgical care [3].

**Fig. 4:**
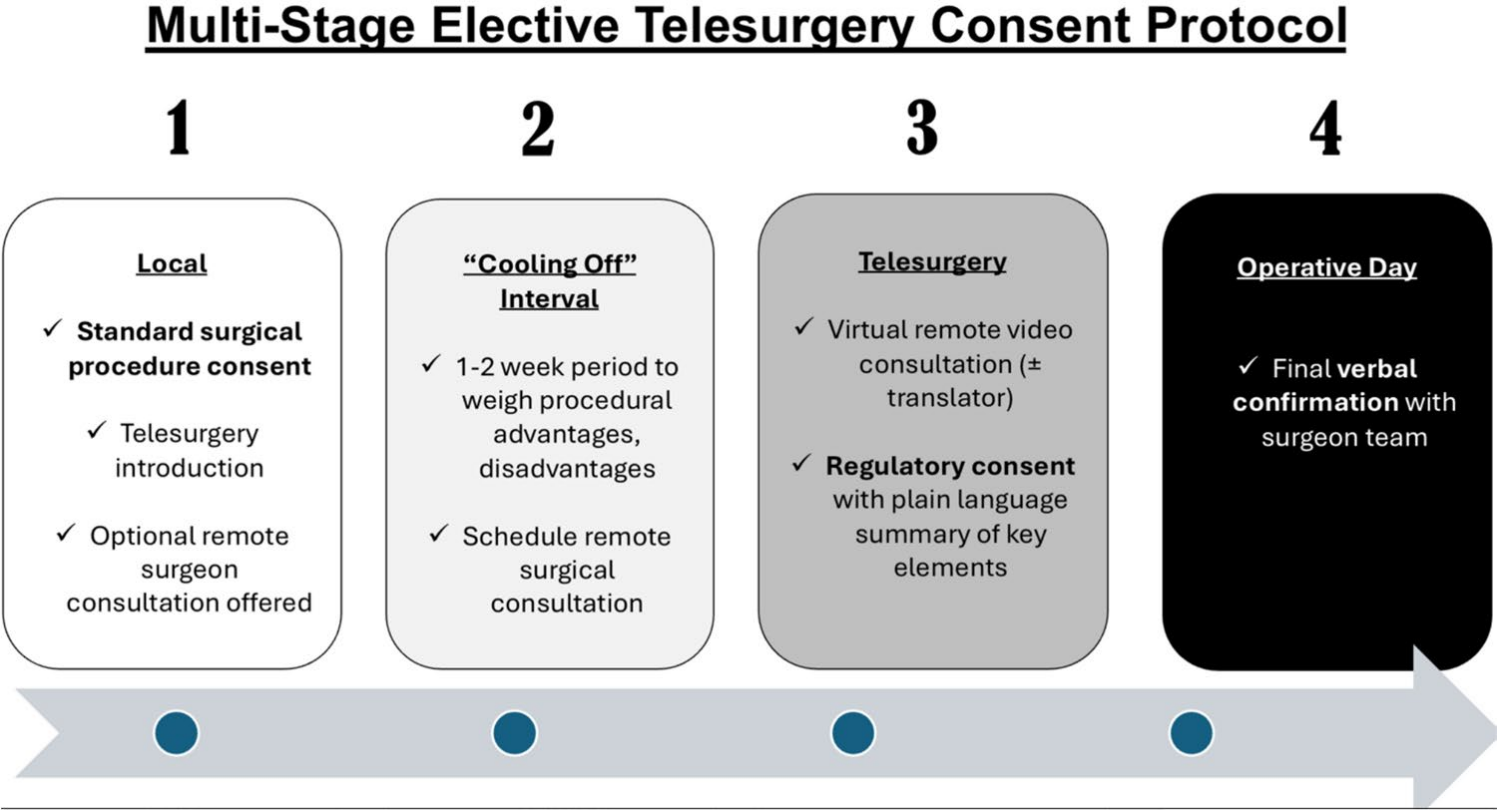
Multi-stage elective telesurgery consent protocol

A multi-stage consent with local and virtual remote surgeon involvement is a practical method to limit patient vulnerability and enhance patient autonomy. This strategy has been historically employed in high-stakes innovative surgical procedures, such as the first living-related liver donors in pediatric liver transplantation [28]. Two-stage consent was proposed by Miller et al. as a strategy to mitigate the ethical challenges in providing high quality IC for surgical innovations. A “cooling off” period between two consent discussions tempers optimism bias by allowing patients to thoughtfully weigh procedural advantages and disadvantages. A multi-stage IC for elective telesurgery may be easily operationalized (Fig. 4). Patients can participate in a standard surgical IC with their local surgical team and then are introduced to the concept of telesurgery. Patient eligibility criteria is screened. Patients should be encouraged to participate in a 15 to 30 minute video conference with the remote surgeon within 1-2 weeks, with translator support as needed. Some time, but not too much time, should pass between stages to optimize information uptake and retention. A second consent/regulatory touchpoint for telesurgery may then ensue. Patient waiver or opting-out of a virtual meeting should be discouraged and the surgical team should consider whether this is disqualifying on an individual basis. A multi-stage consent should be the current best-practice standard for elective telesurgery IC, as it offers the patient the most comprehensive discussion of procedural risks, benefits, and alternatives.

**Fig. 5:**
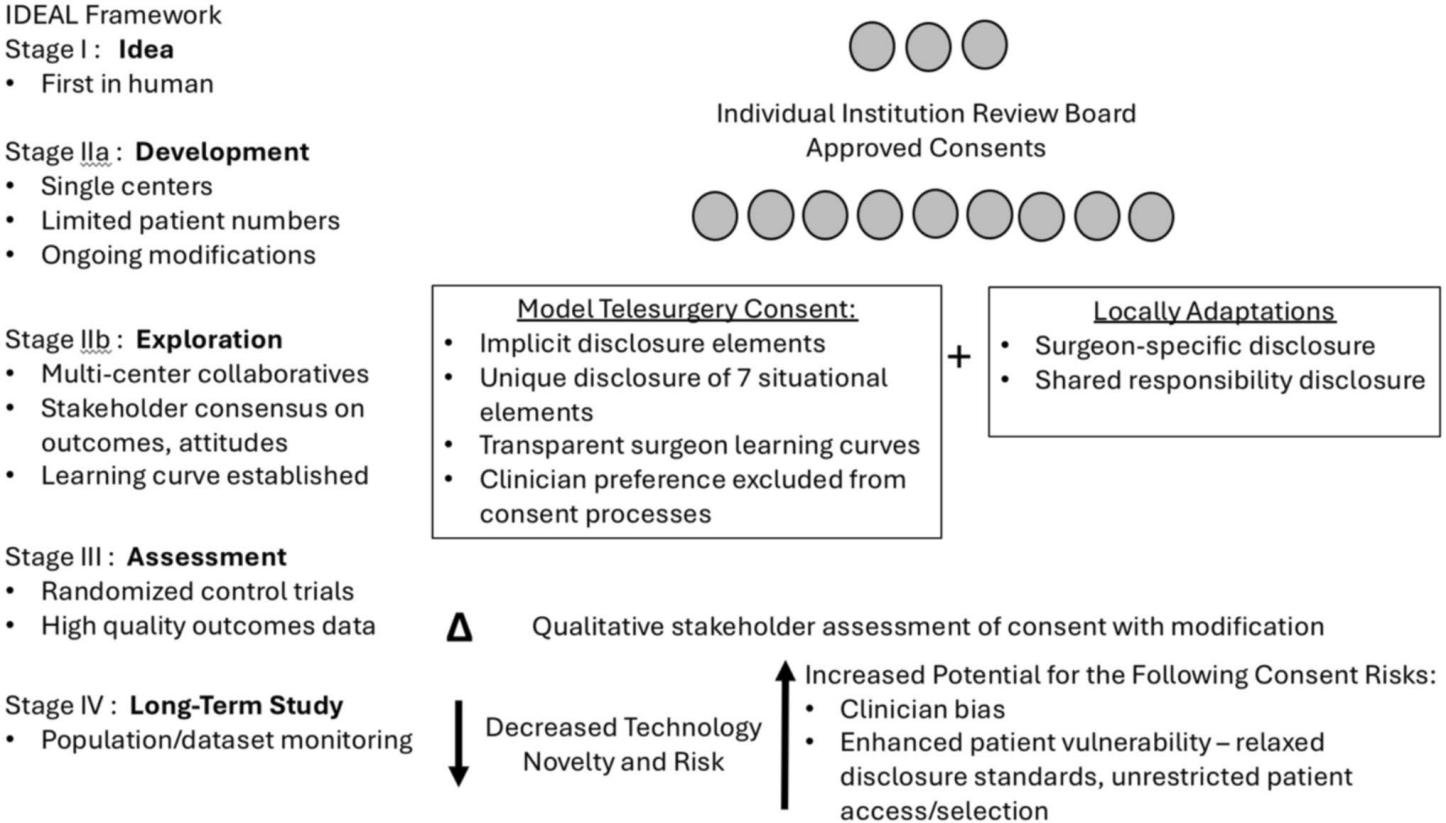
Evolution of telesurgery informed consent through the IDEAL stages of innovation

As telesurgery evolves though the IDEAL innovation process, IC, too, will need to evolve (Fig. 5). The protocols necessary for individual institutions and healthcare systems to perform clinical research during the early idea and development stages of the framework must transform in Stage IIb to streamline multi-center collaboration. The IC process will be refined in the context of stakeholder consensus. At this time, an accepted model for elective telesurgery IC is likely to arrive. This model must consistently uphold core ethical standards, such as through routine disclosure of this framework’s implicit elements and structural consistency in disclosure of the unique situational elements. Accepted models will have to accommodate local heterogeneity in disclosure of shared responsibility and remote surgeon-specific disclosures of skill, experience, limitations and COI. Transparency of surgeon capabilities and their position along the learning curve during IC at this innovation stage is imperative. Formal qualitative analysis of the IC process must also occur. This will capture and quantify patient perception of the IC process, including consent quality, health literacy challenges, or unanticipated ethical concerns, thereby facilitating further IC refinement. Once elective telesurgery advances to IDEAL Stage IV, the phase of long-term study, procedures will occur outside the clinical research regulatory framework. Clinicians will be the sole guaranteer of the IC process. This will introduce an additional ethical challenge, since surgeon preference will be more integrated in IC and the potential for patient persuasion will be greater. Protecting vulnerable patients will be most critical in Stage IV, as standards of disclosure may become relaxed and patient access to the technology becomes unrestricted. At this time, patients will benefit from understanding through IC how their surgeon believes their individual characteristics impact the likelihood of telesurgical success.

It must be emphasized that if a local reasonable alternative to telesurgery does not exist, patients become more vulnerable to persuasion and only an illusion of IC and patient autonomy exists. Patient limitations due to financial status, rurality or dependents that impact travel ability, impaired access to a second opinion or other barriers to quality surgical care may restrict patients from having valid practical alternatives to telesurgery. The unavoidable, inherent global challenge of surgical care disparities is that rural or disadvantaged patients may be a particularly vulnerable group with respect to telesurgery. This degree of patient vulnerability is not unique to telesurgery. Patient vulnerability is generally one of the top four ethical concerns when innovative clinical interventions are introduced [6]. Surgical ethics frameworks are routinely built around the need to protect the most vulnerable patients, including pediatric patients treated with deep brain stimulation for medication-resistant movement disorders, for the psychiatrically vulnerable in need of facial transplantation, or for the critically ill who require extracorporeal membrane oxygenation.

**Fig. 6:**
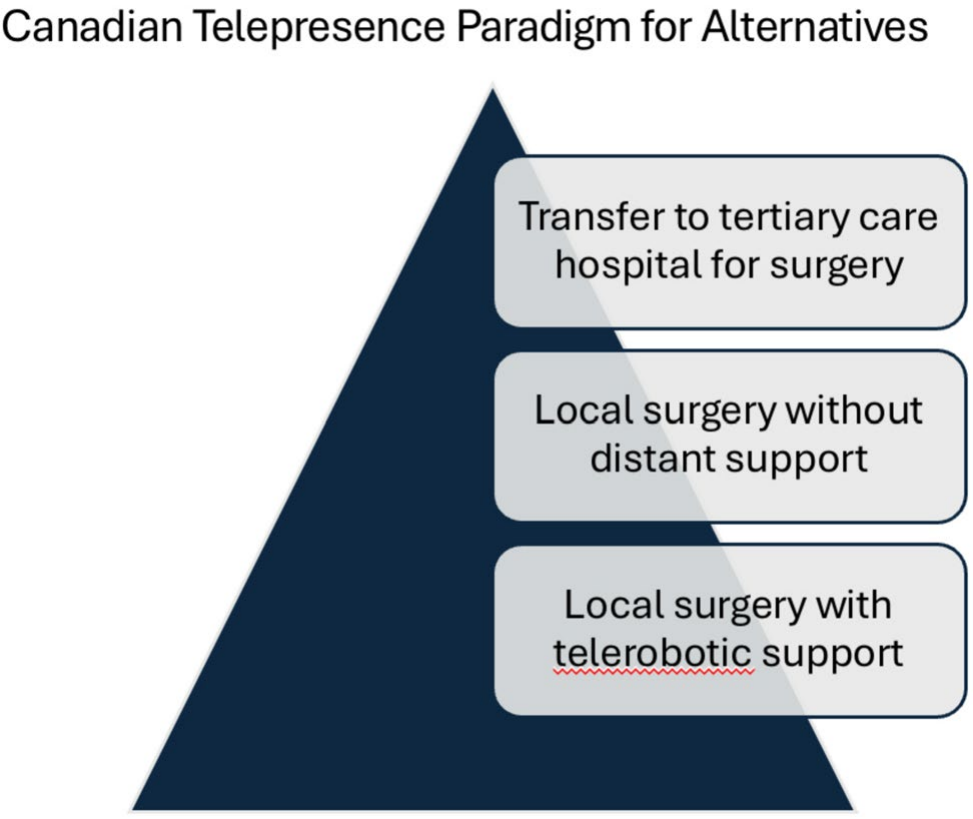
Canadian telepresence paradigm for surgical alternatives

Current telesurgical groups have demonstrated excellence in ensuring local patients have valid alternatives to remote surgery. Examples include the Angola telesurgery project, where robotic radical prostatectomies for cancer are offered to patients in Angola with or without telesurgery, during which a qualified surgeon is at the bedside who has the skills and experience to perform open surgery, if indicated [29]. Another excellent example is the Canadian paradigm published by Anvari et al., where patients were able to select either local laparoscopic surgery with telementoring or local open surgery (Fig. 6) [19]. All patients participating in this trial preferred to access the expertise of a telerobotic surgeon while remaining in their local hospital under the care of their local surgeon. These examples demonstrate the radical power of elective robotic telesurgery to become a force for surgical equity while remaining true to its moral purpose and prioritizing beneficence and non-maleficence.

The Japan Surgical Society and SRS statements discuss surgeon-specific disclosure elements, but they do not explicitly define these elements [12–14]. The proposed framework outlines the 4 most relevant elements (Fig. 3). Disclosure of remote or local surgeon telesurgical experience is highly relevant. An ethical concern is that, if unregulated, this disclosure may also be highly variable. Surgeons must be prepared to uphold their moral obligation of truth-telling. Details about case experience numbers, successes, failures, and complications should be volunteered. There is also a pivotal surgeon obligation to inform the patient if he or she is specifically the first patient to undergo telesurgery in the hands of the local or remote surgeon [28].

Regarding COI, comprehensive disclosure of non-financial COI remains controversial. Patients attribute moral integrity to their surgeons and assume that their surgeon will only offer them standard of care options without disqualifying COI. Surgeons have a moral obligation to uphold best professional practices but may not consistently highlight their parallel academic or financial interests unless specifically asked. Generally, surgeons should recuse themselves if they harbor disqualifying COI (direct atypical financial benefit from patient referrals, ownership of telesurgery infrastructure companies, etc.), but relative COI, such as reputational benefit and academic prestige are rarely disclosed directly [6]. Surgical innovators often display unique phenotypes – they are experts dedicated to “doing good” yet willing to butt heads with the status quo, and they are inherently less risk averse [30]. They may be driven by optimism bias, having a natural desire to implement the procedure they believe to be most beneficial, while forming critical relationships with medical device companies [28]. Core values of honesty and transparency must guide telesurgeon innovators as they collect and report data, maintain professional relationships with industry, and report all relevant financial ties to their institution and patients.

Limitations of this scoping review of the literature and proposed ethics framework for elective telesurgery IC should be acknowledged. A single reviewer assessed the literature. Only articles published in English or with available English translations were considered. The database search was limited to focus on the more prominent scientific databases for physician publications (versus nursing/allied health professionals, or legal professionals). The evidence base (only four documents) is small. The framework proposed is ethics-focused and lacks several important elements present in a formal clinical framework, including validation via multidisciplinary Delphi consensus of relevant stakeholders, pilot testing, patient and stakeholder input. The authors may demonstrate bias due to their narrow geographic and legal perspectives. Additionally, the authorship overlaps with the core evidence base.

## Conclusion

In summary, ethical standards for disclosure during informed consent (IC) for elective telesurgery should include situational, local and remote surgeon-specific elements and address accountability or shared perioperative responsibilities. A local and virtual interactive multi-stage consent process should be offered in an opt-out fashion to minimize patient vulnerability to COI and maximize their autonomy. This proposed multi-stage elective telesurgery IC model can be further adapted regionally. Rather than serve as a rigid standard, it should aim to foster a strong ethical foundation for future telesurgery consent consensus efforts. Surgical consent will naturally vary according to local realities, laws and customs. Discussions that consider best practices regarding COI disclosure should continue. An important ethical concern for the future is the extent to which remote telesurgeons should have obligatory postoperative responsibilities. Failure and complications should continue to be monitored globally, as off trial IC for elective telesurgery is a concept for the future, not yet for “today.”

## Data Availability

No datasets were generated or analysed during the current study.
